# Update on antimicrobial resistance in Somalia: Current status, challenges, opportunities, and future perspectives

**DOI:** 10.1016/j.heliyon.2024.e39434

**Published:** 2024-10-18

**Authors:** Shafie Abdulkadir Hassan, Ahmed Mohamed Dirie, Nur Rashid Ahmed, Abdifetah Ibrahim Omar

**Affiliations:** a Department of Medical Laboratory Sciences, Faculty of Medicine and Health Sciences, Jamhuriya University of Science and Technology, Mogadishu, Somalia; b Faculty of Health Sciences, Salaam University, Mogadishu, Somalia; c Department of Medicine and Surgery, Faculty of Medicine and Health Sciences, Jamhuriya University of Science and Technology, Mogadishu, Somalia; d Jamhuriya Research Center, Jamhuriya University of Science and Technology, Mogadishu, Somalia

**Keywords:** Antimicrobial resistance, Somalia, Challenges, Opportunities, One health

## Abstract

Antimicrobial resistance (AMR) is a critical global health challenge, and Somalia is no exception. This update examines the current status of AMR in Somalia, highlighting the prevalent patterns of resistance, contributing factors, and significant health impacts. Despite limited surveillance data, evidence suggests rising resistance to key antibiotics, exacerbated by inadequate healthcare infrastructure, overuse of antimicrobials, and lack of regulatory oversight. The review identifies key challenges, including insufficient diagnostic capabilities, poor infection control practices, and a need for robust stewardship programs. Opportunities for addressing AMR in Somalia are discussed, including strengthening surveillance systems, improving healthcare access, and fostering international collaboration. Future perspectives emphasize the importance of integrating AMR strategies into broader health policies, enhancing public awareness, and investing in research to develop new treatments and prevention methods. Addressing these issues is crucial for mitigating the impact of AMR and improving health outcomes in Somalia.

## 1 Introduction

Antimicrobial resistance (AMR) is recognized as one of the major public health threats of the twenty-first century [1]. In 2019, AMR was responsible for over 1.2 million deaths worldwide, with Africa and South Asia bearing the highest burden, and this is projected to rise to nearly 10 million deaths annually by 2050 if urgent actions are not taken to address this threat [2]. “In Somalia” the AMR situation is critical, with an estimated 8400 deaths directly attributed to AMR in 2019 and 32,700 deaths linked to AMR-related complications [3]. These figures place Somalia among the top 10 countries with the highest AMR-associated mortality rates globally, surpassing deaths from maternal and neonatal diseases, infectious diseases, cardiovascular conditions, enteric infections, and even certain types of cancers [4].

**Fig. 1:**
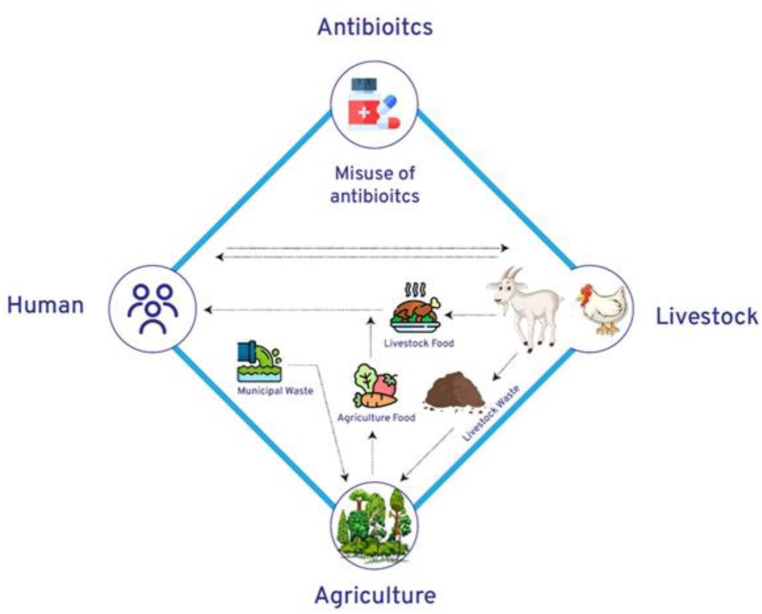
The one health approach to antimicrobial resistance.

AMR occurs when antimicrobial drugs lose their effectiveness and can no longer kill the pathogens, due to misuse and overuse across human, animal, and environmental sectors, coupled with the global spread of resistant microbes [5,6]. Addressing AMR requires a holistic approach that integrates human, animal, and environmental health; known as the One Health, which offers a comprehensive framework for managing AMR more sustainably and effectively, emphasizing the interconnectedness of the health of people, animals, and ecosystems and seeks to optimize the health of all three through coordinated efforts and policies (see Fig. 1) [7].

Somalia presents a unique case of AMR challenges, due to its struggle with prolonged civil unrest and instability, severely disrupting healthcare systems and governance structures, leading to weakened regulatory oversight, poor drug quality control, and irrational prescription practices, resulting in the emergence and spread of AMR [8]. This has resulted in a fragile, fragmented, and weak healthcare system where aid workers are often targeted for carrying out life-saving humanitarian work, consequently, the country's capacity to prevent, detect, and respond to emerging and expanding health threats, such as AMR, has been substantially compromised, necessitating urgent international support and improved governance to address these critical health security challenges [9].

The 2019 Global Health Security Index for Somalia was a mere 16.6 out of 100, indicating the country's severe under-preparedness to tackle AMR, highlighting critical gaps in surveillance, monitoring, laboratory capacity, infection prevention, control measures, antimicrobial stewardship, and policy implementation [10]. These challenges highlight the urgent need for strengthened governance, enhanced healthcare infrastructure, and increased international collaboration to combat AMR in Somalia [10].

This study contributes significantly to the understanding of the antimicrobial resistance by providing a comprehensive analysis of the current status in Somalia, emphasizing the unique challenges faced in this low-resource, conflict-affected country. By highlighting the critical gaps and identifying opportunities for comprehensive interventions, the study thus offers a framework for addressing AMR not only within Somalia but also in other countries facing similar constraints. The insights provided aim to inform evidence-based strategies and policy recommendations for AMR control and management in fragile healthcare systems, reinforcing the importance of a coordinated One Health approach to tackle this growing threat to public health.

### 1.1 Antimicrobial resistance: definition and historical perspective

The history of AMR can be traced back to the discovery of penicillin by Alexander Fleming in 1928 and its mass production in the 1940s [11]. Resistant strains quickly emerged, with the first cases of penicillin-resistant *Staphylococcus aureus* reported in 1942 [12]. Tetracycline resistance followed in 1953, and the widespread use of antibiotics in agriculture in the 1950s and 1960s further accelerated resistance development [13]. The emergence of *methicillin-resistant Staphylococcus aureus* in 1961 and the global epidemic of multidrug-resistant tuberculosis (MDR-TB) in the 1980s highlighted the growing threat posed by AMR [14]. By the 1990s, gram-negative pathogens like *Escherichia coli* and *Klebsiella pneumoniae* developed extended-spectrum beta-lactamase (ESBL) resistance, causing a decline in the effectiveness of available antibiotics and leading pharmaceutical companies to reduce antibiotic research [15].

### 1.2 Current status of AMR in Somalia

The current state of antimicrobial resistance (AMR) in Somalia presents significant challenges; however, the country has taken steps to address this issue by focusing on antibiotic resistance, anti-tuberculosis (TB) efforts, and anti-malaria strategies. This section concentrated on these three key areas, citing recent studies to highlight the progress and ongoing challenges in each area.

### 1.3 Antibiotic resistance in bacterial pathogens

Antibiotic resistance represents a critical public health threat in Somalia, reflecting the alarming global surge in resistant bacterial strains. This section provides an in-depth analysis of the prevalence and resistance patterns among key bacterial pathogens isolated from a range of infections across different patient populations in Somalia. The findings are based on recent studies conducted among various groups, including outpatients, children, and patients with nosocomial infections.

One of the most frequently isolated bacteria in urinary tract infections (UTIs) is *E. coli*. This pathogen is well-documented for its significant role in both uncomplicated and complicated UTIs. The increasing antibiotic resistance of *E. coli* poses a substantial challenge to the effective treatment and management of these infections. Recent studies reveal alarming trends in resistance patterns among *E. coli* strains isolated from various patient populations.

For instance, in a study of outpatients, *E. coli* strains showed high resistance rates to ceftriaxone (97.1 %) and cefixime (61.8 %). Despite these elevated resistance levels, the isolates exhibited moderate susceptibility to ciprofloxacin (67.6 %) and high susceptibility to nitrofurantoin (85.3 %) [16]. In a study focusing on diabetic patients with UTIs, the resistance pattern was even more pronounced, with *E. coli* strains showing complete resistance to cefotaxime (100 %) while colistin (99.2 %) and imipenem (88.3 %) remained the most effective treatment options. However, these isolates also exhibited high resistance to ciprofloxacin (77.5 %) and ofloxacin (90.8 %) [17]. Similarly, a study of catheterized patients with UTIs found that over half of the *E. coli* isolates were resistant to fluoroquinolones, with resistance rates reaching 68.7 % for ciprofloxacin and 52.4 % for levofloxacin. Resistance to cephalosporins was also notably high at 61.6 %, while resistance to carbapenems varied between 7 % and 30 %. Among non-fermenters, *Pseudomonas aeruginosa* demonstrated significant resistance to ceftazidime (53.8 %) but showed lower resistance to other antibiotics. Additionally, *Acinetobacter baumannii* exhibited low resistance to tigecycline and colistin, while *Staphylococcus aureus* had a resistance rate of 16.6 % to vancomycin and linezolid [18].

Beyond *E. coli*, other bacterial pathogens also exhibit troubling resistance patterns. Research on bacterial pathogens in children under five years with diarrhea revealed that all isolates were completely resistant to ampicillin, trimethoprim-sulfamethoxazole, and tetracycline. Among *Shigella species*, *S. flexneri* and *S. sonnei* showed notable resistance to ciprofloxacin and ceftriaxone, with *S. sonnei* exhibiting the highest resistance at 66.7 % [19].

In patients with otitis media, bacterial species such as *Staphylococcus aureus* showed complete resistance to penicillin G and ampicillin, with high resistance to cefotaxime (66.7 %) and erythromycin (72.7 %). *Coagulase-negative staphylococci* were fully resistant to ampicillin and highly resistant to other antibiotics, though they remained susceptible to vancomycin and daptomycin. Pathogens like *Pseudomonas* spp. demonstrated high resistance to cefuroxime (86.7 %) and tetracycline (62.5 %), but lower resistance to meropenem (19.2 %) and piperacillin/tazobactam (7.8 %) [20].

In surgical site infections*, E. coli* and *Klebsiella* spp. showed high resistance to various antibiotics, although resistance to imipenem remained low. *Staphylococcus aureus* was highly resistant to penicillin G and ampicillin but remained susceptible to vancomycin and linezolid [21].

Furthermore, in cases of hospital-acquired pneumonia, different pathogens exhibit varying resistance profiles. *Acinetobacter baumannii* displayed 100 % resistance to many antibiotics, yet remained susceptible to colistin (2.1 %) and tigecycline (4 %). *Pseudomonas aeruginosa* showed high overall resistance, with lower resistance to colistin (4 %) and amikacin (9 %). *Stenotrophomonas maltophilia* demonstrated full resistance to multiple antibiotics, with comparatively lower resistance to ciprofloxacin, levofloxacin, and trimethoprim/sulfamethoxazole (9 %). Notably, high resistance to carbapenems was observed across these pathogens [22] (see Table 1).

### 1.4 Anti-TB drug resistance

The rising prevalence of anti-tuberculosis (TB) resistance in Somalia, particularly multidrug-resistant TB (MDR-TB) and rifampicin-resistant TB (RR-TB) has emerged as a critical public health challenge (see Table 2). These forms of TB complicate treatment regimens and increase the difficulty of controlling the disease.

**Table 1:** Most common antibiotics used and their resistance profiles in different clinical samples.

Sample source	Bacteria	Antibiotic	Resistance (%)	References
Urinary Tract Infections				
	E. coli	Ceftriaxone	97.1	
		Cefixime	61.8	M. A. Mohamed et al. [16]
		Ciprofloxacin	67.6	
		Nitrofurantoin	85.3	Hassan et al. [17]
		Cefotaxime	100	
		Ciprofloxacin	77.5	
		Ofloxacin	90.8	
		Fluoroquinolones	68.7	
Diarrhea				
	S. flexneri	Ciprofloxacin	19.2	
		Ceftriaxone	66.7	
		Trimethoprim-sulfamethoxazole	100	Ali Nor et al. [19]
		Tetracycline	100	
Otitis Media				
	Staphylococcus aureus	Penicillin G	100	
		Ampicillin	100	
		Cefotaxime	66.7	Mohamed Ali et al. [20]
		Erythromycin	72.7	
	CONS	Ampicillin	100	
	Pseudomonas spp.	Cefuroxime	86.7	
		Tetracycline	62.5	
		Meropenem	19.2	
		Meropenem	19.2	
		Piperacillin/tazobactam	7.8	
Surgical Site Infections				
	E. coli	Cefotaxime	93	A. H. Mohamed et al. [21]
	Klebsiella spp.	Imipenem	2.5	
	Staphylococcus aureus	Tetracycline	43.8	
		Ampicillin	97.3	
Pneumonia				
	Acinetobacter baumannii	Colistin	2.1	Adan et al. [22]
		Tigecycline	4.0	
	Pseudomonas aeruginosa	Colistin	4.0	
		Amikacin	9.0	
	Stenotrophomonas maltophilia	Ciprofloxacin	9.0	
		Levofloxacin	9.0	
		Trimethoprim/sulfamethoxazole	9.0	
		Carbapenems	92

**Table 2:** Anti-TB resistance status in Somalia.

Study	Rifampicin-Resistant TB (%)	MDR-TB (%)	Poly-Drug Resistance (%)	Extensive Drug Resistance (%)
M. A. Mohamed et al., [23]	13.3 %	13.3 %	–	–
Guled et al. [24]	51 %	51 %	–	–
Dirie et al. [26]	10.56 %	1.96 %	0.12 %	0.06 %
M. M. Ali et al. [25]	35 %	–	–	–

Mohamed et al. (2023) conducted a study that identified 714 positive GeneXpert-MTB results. Among these, 86.7 % were drug-susceptible, while 13.3 % exhibited rifampicin resistance, defining them as MDR-TB cases [23]. A study by Guuleed et al. (2016) examined 138 suspected MDR-TB cases, finding that 51 % of these patients had rifampicin-resistant TB [24]. A similar study by Ali et al. (2022) studied 370 presumptive TB suspects, of which 17 % were confirmed to have *Mycobacterium tuberculosis*. Among these cases, a concerning 35 % showed rifampicin resistance [25].

Furthermore, Dirie et al. (2022) focused on 1732 pulmonary TB cases diagnosed by the GeneXpert MTB/RIF test and found that 10.56 % of the cases were drug-resistant, with MDR-TB making up 1.96 %, poly-drug resistance (PDR) at 0.12 %, and extensive drug resistance at 0.06 % [26].

Overall, these studies reveal a significant prevalence of anti-TB resistance in Somalia, with MDR-TB and RR-TB. These findings underscore the urgent need for targeted interventions and improved TB management strategies to address the growing challenge of drug-resistant TB in Somalia.

### 1.5 Antimalarial drug resistance

Somalia is a country where *Plasmodium falciparum* is the predominant species, accounting for more than 99 % of infections. In recent years, the spread of *Anopheles Stephensi* in Somalia has been notable. The first detection of *Anopheles stephensi* in the region occurred in Bosasso, Puntland, in 2019 [27]. This was followed by further detections in 2020 across several locations in Somaliland, including Berbera, Hargeisa, and Lawyado, and continued to expand, with reports in 2022 confirming its presence in Burao and Borama, also in Somaliland [28].

Genetic analyses of *Anopheles Stephensi* populations in Somalia have revealed significant findings. Notably, three distinct cytochrome oxidase I (COI) haplotypes have been identified, indicating a certain level of genetic diversity within these populations. Additionally, the detection of the kdr L1014F mutation in these populations is of particular concern, as this mutation is linked to pyrethroid resistance [28]. This resistance could potentially impact malaria control efforts, highlighting the need for ongoing surveillance and adaptive management strategies.

Somalia reported the emergence of chloroquine-resistant *P. falciparum* in 1986. Similarly, resistance to sulfadoxine-pyrimethamine (SP) emerged in the country between 2011 and 2015, shortly after its introduction, necessitating a shift in treatment protocols to artemether-lumefantrine (AL).

In response to rising treatment failures, Somalia has revised its national malaria treatment policy multiple times. In 2005, the country discontinued chloroquine as the first-line therapy for uncomplicated *P. falciparum* malaria, citing high treatment failure rates ranging from 76.5 % to 88 % in regions such as Janale, Jamame, and Jowhar [29]. In 2016 and 2017, the country switched from using Artesunate-SP as a combination therapy to using Artemether-lumefantrine (AL) as the first-line therapy and Dihydroartemisinin-piperaquine (DHA/PPQ) as the second-line therapy. This was done because SP failed over 10 % of the time as a treatment [30].

Somalia has managed to make significant strides in reducing the prevalence of malaria, from 20.1 % in 2015 to 4.1 % in 2023, particularly in the most affected areas [31]. The WHO's strong support for drug resistance containment and malaria elimination activities, along with the adoption of an integrated disease response, have contributed to this progress. The high-level political commitment from Somalia's Ministry of Health and support from international bodies like WHO and Global Fund grants have been crucial in pushing the country towards malaria elimination.

**Fig. 2:**
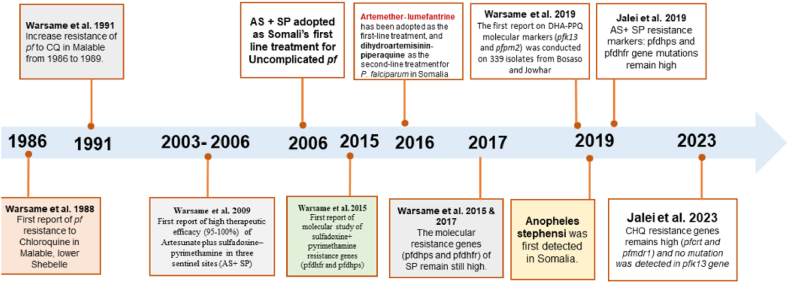
Timeline of malaria treatment evolution and drug resistance in Somalia (1986–2023).

**Fig. 3:**
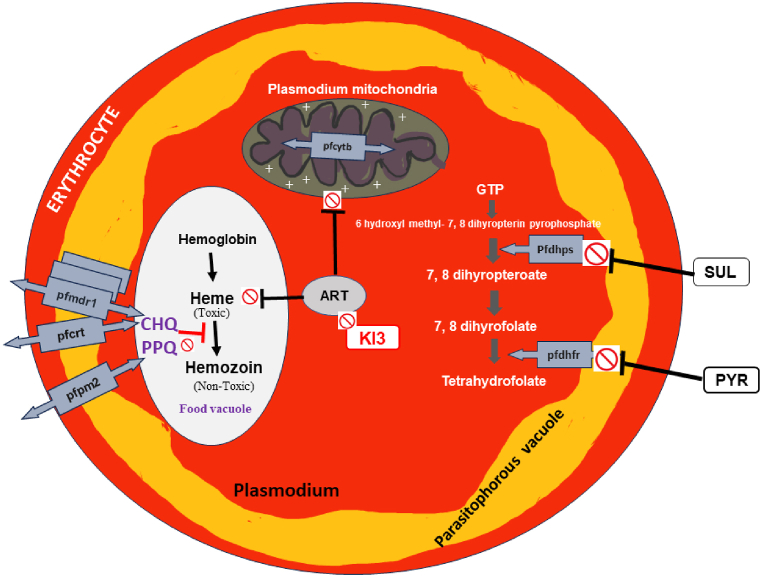
Mechanisms of drug resistance in Plasmodium falciparum: Molecular targets and pathways.

Despite these successes, Somalia continues to face challenges in managing antimalarial drug resistance. Studies conducted in various regions of Somalia, including Jamame, Janale, Jowhar, Bosaso, Afgoi, and Bal'ad, have revealed significant resistance to commonly used antimalarial drugs (Fig. 2). For example, Warsame et al. (2015) and (2017) found that mutations in the dhfr, dhps, pfdhps, and pfdhfr genes were linked to high treatment failure rates in SP and AL. Strong links between these mutations and resistance and therapeutic failures underscore the need for continuous monitoring and strategic approaches to malaria treatment [30,32]. The molecular mechanism of Plasmodium falciparum resistance in Somalia is summarized (see Fig. 3).

Also, Warsame et al. (2019) found that DHA/PPQ and AL have remained effective with no signs of resistance. Other studies, such as those by Jalei et al. (2019) and (2023), show that resistance to SP and chloroquine sensitivity have not reemerged yet, despite the discontinuation of these drugs more than a decade ago. Mutations in the pfcrt and pfmdr1 genes remain prevalent, particularly in isolates from regions like Afgoi, although no mutations have been found in the pfk13 gene, indicating that artemisinin-based therapies still retain their efficacy [33].

### 1.6 Challenges

Antimicrobial resistance (AMR) is a growing global concern, and Somalia faces unique challenges in addressing this issue. The country's specific socio-economic, healthcare, and regulatory environments contribute to the misuse of antimicrobials, driving the spread of resistant pathogens. Below are the key challenges related to AMR in Somalia.

Compounding this issue is the unregulated market and easy access to antibiotics in Somalia, where pharmacies and informal drug vendors operate with minimal oversight, allowing antibiotics to be purchased without a prescription [34].This lack of regulation fosters inappropriate use of antibiotics for conditions that do not require them, which drives antimicrobial resistance (AMR) and highlights the critical challenge of addressing widespread antibiotic availability without proper controls to combat the growing AMR crisis.

A significant challenge in Somalia is the lack of public awareness regarding the appropriate use of antibiotics and the dangers of AMR. Many people in the country do not fully understand that antibiotics are ineffective against viral infections or the importance of completing a full course of treatment [35]. This lack of knowledge leads to harmful practices, such as using leftover antibiotics or discontinuing treatment prematurely, which contribute to the emergence of resistant bacteria. Public education and awareness campaigns are urgently needed to change these behaviors and reduce the spread of AMR.

Limited access to healthcare, especially in rural and conflict-affected areas, forces people to use antibiotics without proper diagnosis, leading to inappropriate use and resistance development [36]. Improving healthcare infrastructure and diagnostic services is crucial, as laboratory capacity issues further hinder effective AMR surveillance and limit timely, accurate data for public health responses.

Economic factors contribute to antimicrobial misuse in Somalia. High healthcare costs relative to income discourage professional medical advice, leading people to buy antibiotics directly from pharmacies or informal vendors [37]. This financial constraint and the perception of antibiotics as a quick fix result in inappropriate use and the spread of AMR.

Federalism in Somalia impacts healthcare governance by decentralizing authority, leading to challenges in resource allocation for antimicrobial resistance (AMR) and the insufficient funding creates gaps in surveillance, diagnostics, and public awareness, compromising AMR management [38].

The absence of effective regulations and surveillance in both human and animal health sectors, coupled with limited access to quality healthcare and inadequate monitoring systems, significantly contributes to the spread of AMR by allowing misuse and overuse of antibiotics and hindering timely diagnosis and appropriate treatment [8,39].

Furthermore, food production in Somalia faces critical challenges, including practices like open-air animal slaughter that lead to the contamination of rivers used for drinking water. This contamination increases the risk of antimicrobial resistance (AMR) as resistant bacteria from animal waste can spread into the water supply [8]. Somalia lacks a surveillance system to monitor antibiotic use in food production and track AMR in food processing.

### 1.7 Opportunities

Somalia faces significant challenges in managing antimicrobial resistance (AMR) due to antibiotic misuse, inadequate healthcare infrastructure, and gaps in surveillance capabilities, yet there are promising opportunities to improve health outcomes by leveraging existing resources and fostering cross-sector collaboration.

One of the most significant opportunities for Somalia lies in enhancing surveillance systems by establishing the National Public Health Laboratory and the National Action Plan on AMR, expanding these systems to cover more regions, integrating data across healthcare facilities, and reinforcing laboratory networks while fostering collaboration between public health and veterinary sectors to enable early detection of resistance patterns and timely, targeted interventions [40].

Reinforcing national policies and guidelines, such as the Somali National Medicines Policy and the Somali National Treatment Guidelines, is crucial for managing antibiotic use and ensuring rigorous implementation and adherence across both public and private healthcare sectors to promote responsible antibiotic use and help mitigate the spread of resistance [41,42].

Improving the efficiency of the medicines supply chain presents a significant opportunity by leveraging the National Medicines Supply Chain Master Plan and the National Medical Warehouse to optimize medicine distribution, ensure the availability of quality-assured antibiotics, reduce risks associated with counterfeit drugs, and support appropriate antibiotic use.

Integrating AMR-focused training into professional education represents a valuable opportunity, as the National Health Professional Council can oversee healthcare standards and drive the inclusion of AMR modules into medical, nursing, and veterinary curricula, while ongoing professional development will ensure healthcare professionals are equipped with the latest knowledge and skills to manage AMR effectively.

International collaboration offers substantial benefits, as partnerships with organizations like WHO and participation in global AMR networks provide Somalia with access to technical expertise, funding, and best practices, and strengthening these international ties will enhance Somalia's capacity to implement effective AMR strategies and interventions while benefiting from global experiences and resources.

The integration of digital health technologies is a critical opportunity, as implementing electronic health records (EHRs) and digital surveillance tools will streamline the collection, analysis, and dissemination of AMR data, supporting better clinical decision-making and more effective management of antibiotic use while improving overall healthcare delivery.

Engaging the private sector is essential for a comprehensive approach to AMR, as private healthcare providers and pharmaceutical companies can play a crucial role in surveillance, reporting, and educational efforts, enhancing AMR practices, ensuring the availability of quality medicines, and supporting the development of innovative treatments.

**Fig. 4:**
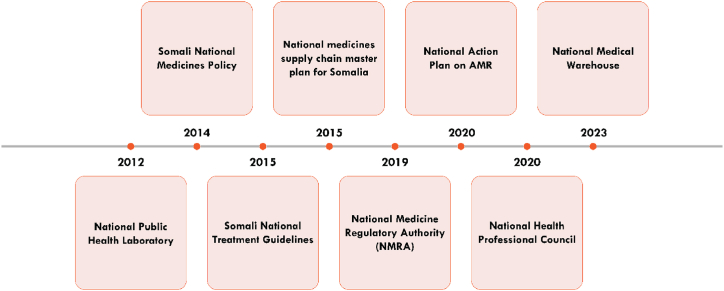
Strategic actions and developments in public health over 10 Years.

By capitalizing on these opportunities and fostering collaboration among government agencies, international organizations, healthcare providers, and the private sector, Somalia can significantly strengthen its approach to AMR, with effective resource utilization, infrastructure investment, and active public engagement being key to reducing AMR and safeguarding public health. The summary of actions and developments related to public health in Somalia over the last 10 years is presented (see Fig. 4).

### 1.8 Future perspectives

Given the growing threat of antimicrobial resistance (AMR) and its severe impact on health outcomes in Somalia including increased treatment failures, rising healthcare costs, and additional strain on an already overburdened healthcare system, it is crucial to focus on the following areas:

Strengthening political commitment and governance is crucial, as strong political will is vital for AMR initiatives to succeed; the government should fully implement and continuously update the National Action Plan (NAP) on AMR to align with international standards, enhance coordination between federal and regional health authorities, and secure long-term funding through national budgets and international aid to sustain AMR activities [43].

Enhancing surveillance and data management is fundamental, as establishing an integrated national surveillance system across human, animal, and environmental health sectors will improve AMR management while investing in laboratory infrastructure, training personnel, and utilizing digital health technologies will enhance the reliability and efficiency of data collection and reporting [44].

Promoting the rational use of antimicrobials is crucial, with strict regulations on antibiotic prescription and sale, nationwide public awareness campaigns about misuse, and implementing antimicrobial stewardship programs in healthcare facilities to guide appropriate use and reduce unnecessary prescriptions [45].

Investing in healthcare infrastructure and capacity building is vital for effective AMR control, as expanding and upgrading facilities, particularly in remote areas, enhances access to quality care and timely diagnosis, while continuous education and training on AMR and antimicrobial stewardship for healthcare workers, including veterinarians, are essential, and supporting veterinary services to monitor and control antimicrobial use in animals helps prevent the spread of resistance to humans [46].

International collaboration and partnerships can provide valuable expertise and resources, as strengthening ties with global health agencies, research institutions, and NGOs offers technical support and funding, while participation in international AMR networks facilitates knowledge sharing and best practices, and public-private partnerships leverage additional resources [47].

Strengthening public awareness and community engagement is necessary to combat AMR, with targeted educational campaigns and community-driven initiatives raising awareness and promoting responsible antibiotic use while engaging community leaders and patient advocacy groups enhance public involvement in AMR initiatives [35].

Supporting research and development is crucial for finding innovative solutions to AMR, as funding research on new antibiotics, alternative treatments, and rapid diagnostic tools will drive innovation, with collaborations between academic institutions, research centers, and the pharmaceutical industry being essential for developing effective AMR interventions tailored to Somalia's unique challenges [48].

Looking ahead, addressing antimicrobial resistance (AMR) through a One Health approach is crucial due to the interconnected nature of human, animal, and environmental health This integrated and unified strategy combines various disciplines and sectors to tackle these challenges effectively.

Additionally, improving nutrition may play a crucial role in reducing susceptibility to diseases that often require antibiotic treatment, as enhanced nutritional status can strengthen immune responses and lower infection rates, potentially decreasing reliance on antibiotics and bolstering efforts to combat AMR by promoting better health and resilience across populations [49].

Finally, using alternative treatments like traditional remedies and medicinal plants instead of conventional antibiotics is crucial for reducing antimicrobial resistance (AMR) At the same time, other options such as vaccines, antibodies, pattern recognition receptors, probiotics, bacteriophages, peptides, phytochemicals, metals, and antimicrobial enzymes are being explored to further reduce our reliance on antibiotics [50].

By implementing these recommendations and considering the role of nutrition, Somalia can develop a comprehensive and sustainable approach to managing antimicrobial resistance, ultimately safeguarding public health and addressing the growing threat of AMR.

## 2 Conclusion

Addressing antimicrobial resistance (AMR) in Somalia is crucial for safeguarding public health. The rising rates of AMR are driven by inadequate surveillance, overuse of antibiotics, and weak infection control. To combat these challenges, it is essential to enhance diagnostic capabilities, strengthen healthcare infrastructure, and foster international collaboration. Additionally, exploring alternative treatments and incorporating effective nutritional strategies can play a significant role in managing infections and reducing reliance on antibiotics. Promoting research into new therapies and preventive measures, as well as integrating AMR strategies into broader health policies, will further bolster efforts to improve health outcomes and contribute to global AMR combat strategies.

## CRediT authorship contribution statement

**Shafie Abdulkadir Hassan:** Writing – review & editing, Writing – original draft, Visualization, Supervision, Project administration, Conceptualization. **Ahmed Mohamed Dirie:** Writing – review & editing, Visualization, Resources. **Nur Rashid Ahmed:** Writing – review & editing, Visualization, Resources, Project administration. **Abdifetah Ibrahim Omar:** Writing – review & editing, Visualization, Resources, Project administration.

## Data availability statement

This study is not applicable because it does not involve any datasets generated or analyzed.

## Ethics approval

This study is not applicable because it did not involve any human participants or animals.

## Consent to participate

This study is not applicable because it does not involve human participants.

## Consent for publication

This study is not applicable because it does not involve individual participants' data.

## Funding

No funding was received for this study.

## Declaration of competing interest

The authors declare that they have no known competing financial interests or personal relationships that could have appeared to influence this work.
